# Colitis-induced upregulation of tumor necrosis factor receptor-2 (TNFR2) terminates epithelial regenerative signaling to restore homeostasis

**DOI:** 10.1016/j.isci.2023.107829

**Published:** 2023-09-04

**Authors:** Zohreh Sharifkhodaei, Cambrian Y. Liu, Nandini Girish, Ying Huang, Shivesh Punit, M. Kay Washington, D. Brent Polk

**Affiliations:** 1 Department of Pediatrics, University of California San Diego, San Diego, CA, USA; 2 Department of Medicine, The University of Chicago, Chicago, IL, USA; 3 The Saban Research Institute, Division of Pediatric Gastroenterology, Hepatology Nutrition, Children’s Hospital Los Angeles, Los Angeles, CA, USA; 4 Department of Pathology, Vanderbilt University Medical Center, Nashville, TN, USA

**Keywords:** Physiology, Molecular biology, Immunology, Cell biology, Omics, Transcriptomics

## Abstract

Colonic epithelial repair is a key determinant of health. Repair involves changes in epithelial differentiation, an extensive proliferative response, and upregulation of regeneration-associated “fetal-like” transcripts, including *Ly6a* (Sca-1), that represent Yap1 and interferon targets. However, little is known about how this regenerative program terminates and how homeostasis is restored during injury and inflammation. Here we show that, after the initial entry into the regenerative state, the subsequent upregulation of tumor necrosis factor (TNF) receptor 2 (R2, TNFR2, *Tnfrsf1b*) clears the regenerative signaling and restores homeostatic patterns of epithelial differentiation. Targeted deletion of epithelial TNFR2 *in vivo* and in colonoid cultures revealed persistent expression of *Ly6a*, hyperproliferation, and reduced secretory differentiation. Moreover, mice lacking epithelial TNFR2 also failed to complete colon ulcer healing, suggesting that partial resolution of regenerative signaling is essential for the completion of the repair process. These results demonstrate how epithelial cells dynamically leverage a colitis-associated cytokine to choreograph repair.

## Highlights

• Epithelial induction of TNFR2 halts regenerative signaling to induce wound healing
• TNFR2-ablated epithelium fails to complete colonic ulcer healing
• TNFR2 loss leads to reduction of differentiation and persistence of re-programming
• TNFR2 is critical to restore homeostatic function after colonic epithelial injury

## Graphical abstract

**Figure:**
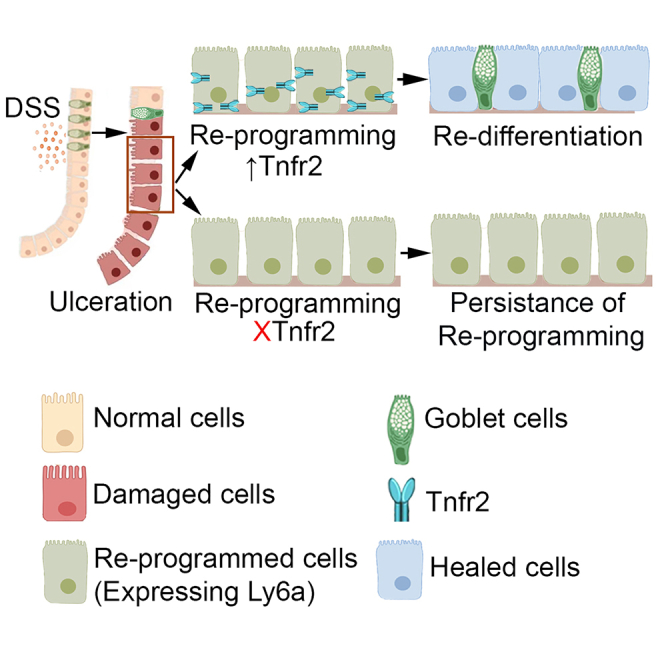

## Introduction

Colonic epithelial repair is an important predictor of positive long-term outcomes in inflammatory bowel disease (IBD) patients, who suffer from chronic injury to the gastrointestinal tract. A deeper understanding of wound healing mechanisms is therefore of therapeutic significance. Recent investigations of wound-induced signaling have highlighted key epithelial mechanisms that initiate healing. Wounded epithelial cells downregulate markers of homeostatic terminal differentiation (e.g., mucins) and stem cell maintenance (e.g., *Lgr5*) while upregulating interferon-associated “fetal-like” transcripts, in a process generally referred to as reprogramming. The regenerative state is marked by expression of *Ly6a* (Sca-1), and reprogramming requires Yap/Taz signaling and potentially other microenvironmental factors.^1^^,^^2^^,^^3^^,^^4^ During repair, progenitor cells of reserve, revival, or dedifferentiated origin rebuild the epithelium. While it is sensible that entry into the regenerative state drives initial wound repair, it is much less clear whether the reverse change—from regenerative signaling to homeostasis—contributes significantly to wound healing.

Although cytokines such as tumor necrosis factor (TNF) are historically associated with regulation of the immune response, they can also directly modulate intestinal/colonic epithelium.^5^^,^^6^ Their pleiotropic effects may seem paradoxical. Elevations in colonic mucosal and serum TNF have been demonstrated in IBD patients,^7^^,^^8^^,^^9^ and reduction of TNF signaling represents one tool in the modern medical management of IBD.^10^^,^^11^ However, TNF signaling also promotes epithelial cell proliferation and stem cell renewal, key parameters of mucosal regeneration.^12^^,^^13^^,^^14^^,^^15^^,^^16^^,^^17^ Moreover, elimination of TNF signaling exacerbates disease in mice and may induce new-onset IBD in humans.^18^^,^^19^^,^^20^^,^^21^^,^^22^ Whether the context-specific functions of TNF are driven by differential activation or upregulation of its receptors remains to be fully elucidated.

Here, we tested whether the dynamic regulation of TNF receptor 2 (TNFR2, encoded by the *Tnfrsf1b* gene), the second of two primary transmembrane receptors of TNF, directs the pattern of regenerative signaling after acute injury and inflammation of the colonic epithelium. Unlike the constitutively expressed TNFR1, which has been classically associated with pro-inflammatory responses in immune cells,^23^ TNFR2 is expressed only on certain subsets of cells such as CD8^+^ T cells.^24^^,^^25^ It is upregulated in intestinal injury and inflammation, including in human Crohn disease.^23^^,^^26^ This induction is partly regulated by high levels of TNF and IL-6.^23^ TNFR2 has been shown to have anti-inflammatory functions in a variety of organ systems. In the intestinal epithelium, TNFR2 positively regulates cell migration and proliferation.^12^^,^^16^^,^^17^^,^^27^ In this report, we characterize the dynamic expression and function of TNFR2 specifically in wound-associated colonic epithelium using deep 3D imaging^28^^,^^29^ and transcriptomic analyses. These results suggest that epithelial upregulation of TNFR2 in late repair halts epithelial regenerative signaling and restores homeostatic secretory cell populations.

## Results

### Colonic epithelial induction of *Tnfr2* in colitis models

**Figure 1:**
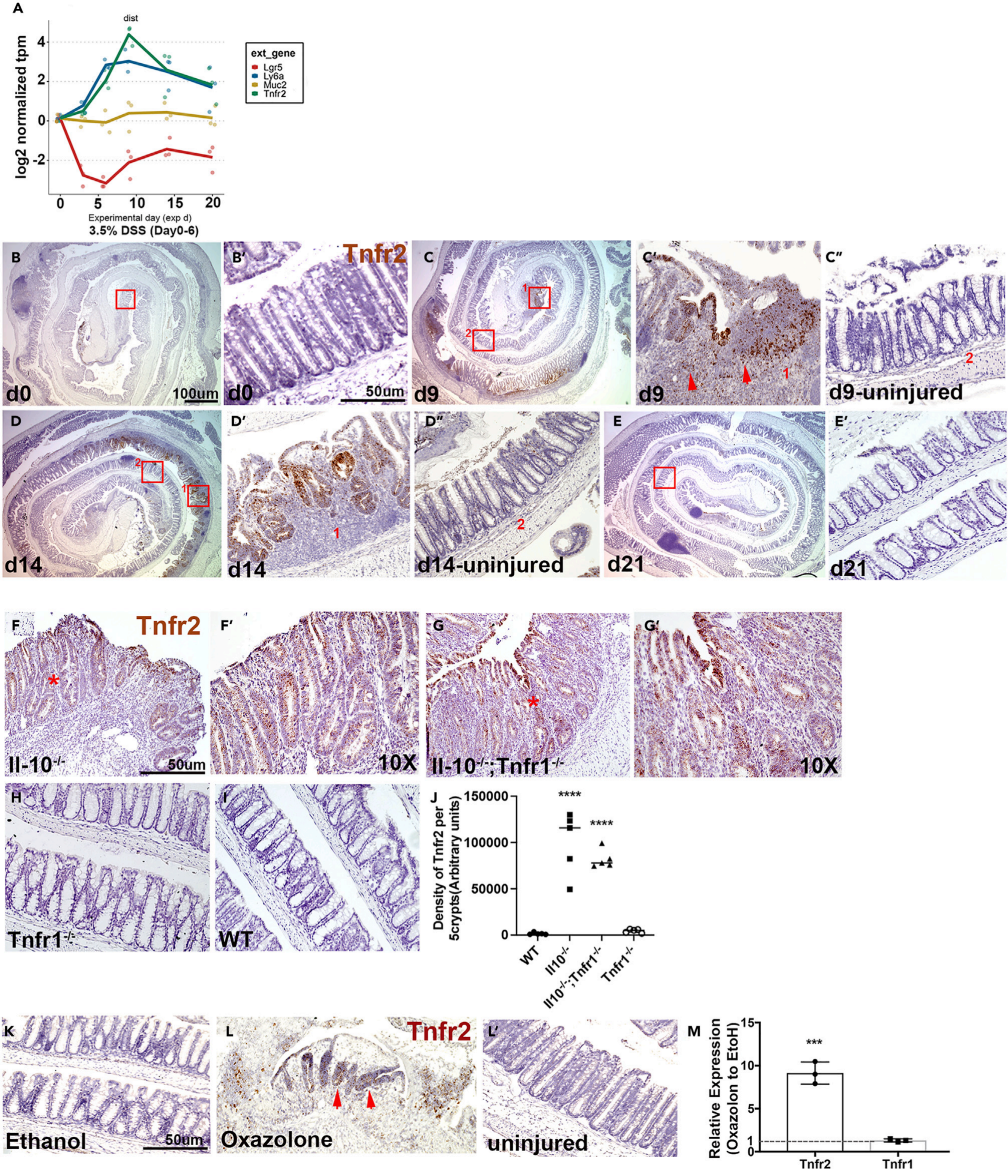
Colonic epithelial induction of Tnfr2 in colitis models Mice (8 weeks, n = 3/group) were exposed to 3.5% DSS water for 6 days followed by regular drinking water to induce DSS colitis injury model. (A) RNA profiling of colonic tissue at various timepoints during DSS-induced colitis showed the significant upregulation of *Tnfr2* transcript at day 9 (∗∗∗p = 0.0001), downregulation of *Lgr5*, induction of *Ly6a*, and depletion of *Muc2*^+^ secretory cells. (B–E) *In situ* hybridization with *Tnfr2* targeted probes showed high levels of Tnfr2 expression in distinct groups of epithelial cells adjacent to the injured region (day 9–14, brown; C′, D′) compared to uninjured crypts of the same tissue (C”, D″) and control group (day 0, B′). (E′) *Tnfr2* level is reduced at day 21. (B, C, D, E) The zoom-out views of Swiss roll colons. Red squares with assigned numbers show the location of zoom-in images. Scale bars: 50 μm,100 μm. (F–J) *In situ* hybridization with *Tnfr2* targeted probes showed the induction of *Tnfr2* in distal colon of *Il-10*^*−/−*^ (F, brown) and *Il-10*^*−/−*^*; Tnfr1*^*−/−*^ (G, brown) compared to *Tnfr1*^*−/−*^ (H) and WT control (I). F′ and G′ showed the zoom-in view of selected area (star) in F and G. (J) The density of *Tnfr2* signal was significantly high in *Il-10*^*−/−*^ and *Il-10*^*−/−*^*; Tnfr1*^*−/−*^ compared to *Tnfr1*^*−/−*^ and WT. (∗∗∗∗p < 0.0001; n = 5, mean ± SD, one-way ANOVA). Scale bar: 50um. (K–M) *Tnfr2* transcript is induced in oxazolone colitis. (K-L) *In situ* hybridization with *Tnfr2* targeted probes showed high levels of *Tnfr2* in epithelial cells adjacent to the injured epithelium (L, brown; arrow) compared to uninjured crypts of the same colon (L′) and Ethanol-treated control (K). (M) qPCR analysis showed the upregulation of *Tnfr2* compared to *Tnfr1* in colonic epithelium. (∗∗∗p = 0.0005; n = 3, mean ± SD, unpaired t test). Scale bar:50 μm.

We assessed the regulation and role of TNFR2 primarily in the dextran sulfate sodium (DSS)-induced model of murine acute colitis. In this model, 8-week-old mice were exposed to 3.5% DSS drinking water for 6 days, beginning on experimental day (d) 0. When this model is performed in our laboratory, ulceration in colonic epithelium typically peaks at d 8–9, followed by gradual healing over a period of 2–4 weeks^29^ Crucially, the induction of *Ly6a* expression, loss of the *Lgr5* stem cell marker, and depletion of secretory cell differentiation are observed in epithelial cells in the distal colon by d 6.^1^^,^^2^ Previous work has suggested that TNFR2 may be upregulated in IBD, although some of this signal may have been associated with mucosal infiltration of immune cells.^23^^,^^26^ Retrospective analysis of RNA-seq data collected from the colonic mucosa at various time points during DSS-induced colitis validated the loss of *Muc2* and *Lgr5* early (d 3–6) during DSS exposure, followed by the upregulation of *Ly6a* (d 6) and peak expression of *Tnfr2* at d 9 (Figure 1A). Thus, mucosal samples demonstrate increased *Tnfr2* expression in association with injury-induced changes in epithelial gene expression.

If TNFR2 were to have unique functions during colonic epithelial wound healing, we reasoned that it should be specifically expressed in epithelial cells near the wound margin. We therefore tested this hypothesis using *in situ* expression profiling of *Tnfr2* transcripts in the colon. Consistent with RNA-seq data, *Tnfr2* expression was very low at baseline in the uninjured colon. However, at time points of significant injury (e.g., d 9 and 14), *Tnfr2* targeted probes preferentially localized to epithelial cells in proximity to ulcerated wound regions (Figures 1B–1E). Epithelial cells that were not immediately adjacent to an ulcer or region of immune infiltration were not marked by the probe (C″, D″). *Tnfr2* expression in epithelium was less common at d 21. These results are consistent with epithelial upregulation of *Tnfr2* in response to DSS-induced injury.

We further assessed whether epithelial upregulation of Tnfr2 was found in other colitis models. In the *Il10−/−* Th1/Th17-like spontaneous colitis model, in which disease can be exacerbated by co-deletion of TNFR1,^18^ we found upregulation of *Tnfr2* to be associated with colitis in both *Il10−/−* and *Il10−/− Tnfr1−/−* mice (Figures 1F, 1G, and 1J). However, the *Tnfr2* transcript was not detected in uninjured wild type or in *Tnfr1*^*−/−*^ mice (Figures 1H, 1I, and 1J). Thus, the elevation of Tnfr2 was associated with injury and not with compensatory loss of TNFR1 signaling. Likewise, in the oxazolone Th2-like colitis model, we also observed the induction of *Tnfr2* adjacent to the injured regions in the colon while the *Tnfr1* transcript remained unchanged (Figures 1K–1M). Thus, Tnfr2 is highly and specifically upregulated in epithelial cells participating in injury-adapted responses.

### Co-localization of Tnfr2 with regenerative and undifferentiated epithelium

**Figure 2:**
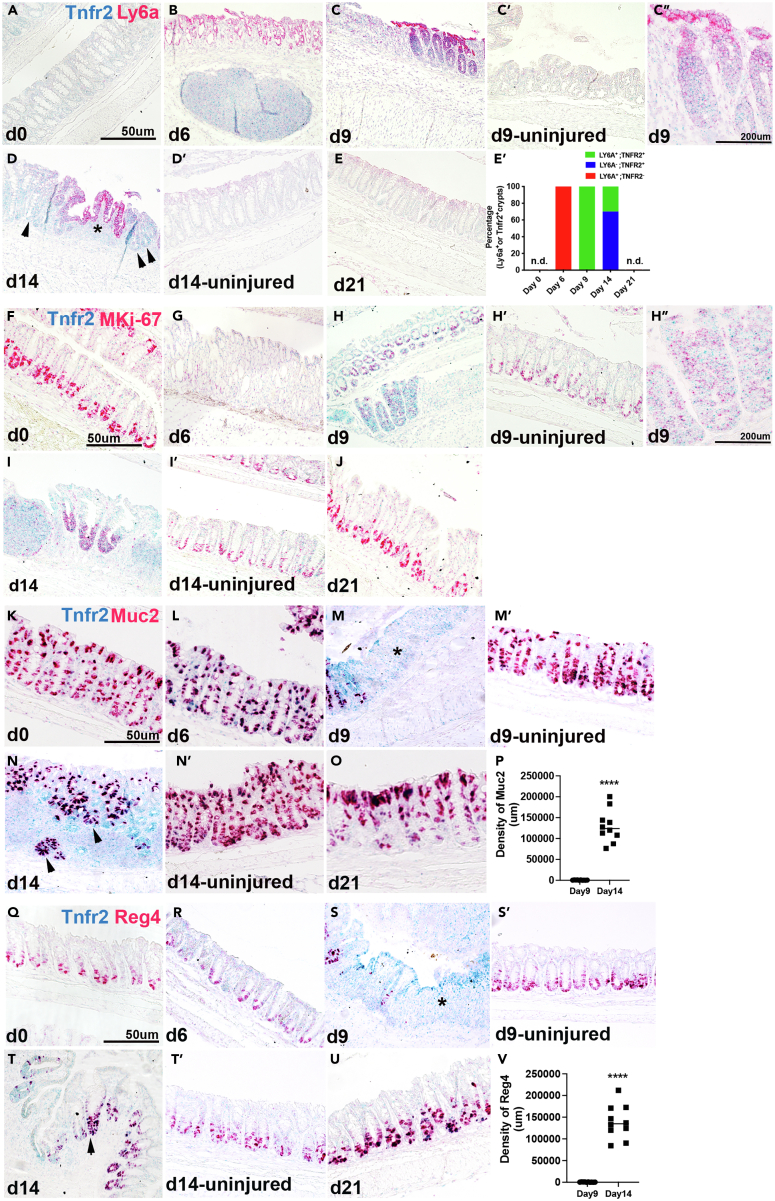
Co-localization of Tnfr2 with regenerative and undifferentiated epithelium ice (8 weeks, n = 3/group) were exposed to 3.5% DSS water for 6 days followed by regular drinking water to induce DSS colitis injury model. (A–E) Duplex *in situ* hybridization with *Tnfr2* (blue) and *Ly6a* (red) targeted probes showed high levels of *Ly6a* at day 6 (B), 9 (C) and 14 (D; star) in injured crypts compared to uninjured areas of the same colon (C′, D′), day 21 (E, recovered tissue) and control (day 0, A). Induced *Tnfr2* transcript was co-localize with *Ly6a* at impaired regions at day 9 (C) and 14 (D; star). The enlarged view of C (d9) is shown in C”. Only Tnfr2 expression was high in newly formed crypts (D; arrows) adjacent to injured crypts (D; star) at day 14. (E′) The fraction of *Ly6a*^*+*^, *Tnfr2*^*+*^, and double-positive crypts at different timepoints was presented in the stacked bar graph. n.d. stands for not detected at day 0 and day 21. (F–J) Duplex *in situ* hybridization with *Tnfr2* (blue) and M*Ki-67* (red) targeted probes showed co-localization of *Tnfr2* with *MKi-67* in injured colonic epithelium at day 9 (H) and 14 (I) compared to uninjured areas of the same colon (H′, I′), day 6 (G), day 21 (J) and control (day 0, F). The enlarged view of H (d9) is shown in H”. (K–P) Duplex *in situ* hybridization with *Tnfr2* (blue) and *Muc2* (red) targeted probes showed lack of *Muc2* expression in injured regions at day 9 (M, star) and co-localization of *Tnfr2* and *Muc2* in new crypts at day 14 (N, arrows) compared to uninjured areas of the same colon (M′, N′), day 6 (L), day 21 (O) and control (day 0, K). (P) Density of *Muc2* transcript was significantly reduced at day 9 and induced at day 14 in TNFR2^+^ cells. (∗∗∗∗p < 0.0001; mean ± SD, unpaired t test). (Q–V) Duplex *in situ* hybridization with *Tnfr2* (blue) and *Reg4* (red) targeted probes showed loss of *Reg4* in injured regions at day 9 (S, star) and co-localization of *Tnfr2* and *Reg4* in new crypts at day 14 (T, arrows) compared to uninjured areas (S′, T′), day 6 (R), day 21 (U) and control (day 0, Q). (V) Density of *Reg4* was significantly reduced at day 9 and induced at day 14 in TNFR2^+^ cells (∗∗∗∗p < 0.0001; mean ± SD, unpaired t test). Scale bars:50 μm, 200 μm.

The emergence of a regeneration-associated cell population expressing *Ly6a* (Sca-1) is a key early event during wound healing. The relationship of the *Ly6a+* cell population to the cells that upregulate *Tnfr2* is not known. We studied the relationship of *Tnfr2* with cells that express markers of regeneration (*Ly6a*), proliferation (*Mki67*), and differentiation (goblet cells, *Muc2*; deep crypt secretory cells, *Reg4*) at different time points after exposure to DSS. Duplex *in situ* hybridization showed the marked elevation of *Ly6a* at the onset of colonic injury (d 6); however, at this early time point, the expression of *Tnfr2* was not detected (Figures 2A, 2B, and 2E′). At d 9, the induction of *Tnfr2* transcript perfectly co-localized with the *Ly6a+* crypts adjacent to inflamed epithelial ulcers (Figures 2C and 2E′). By d 14, the expression pattern of *Ly6a* and *Tnfr2* began to diverge. Some crypts retained positive expression for both markers; however, the clusters of crypts at the base of “wound channels,” indicative of newly formed crypts,^29^^,^^30^ were singly positive for *Tnfr2* (Figure 2D; arrows, 2E′). Epithelial cells away from the injured regions were not marked by either probe (Figures 2C’ and 2D′). Both *Ly6a* and *Tnfr2* levels were reduced at d 21 when the mucosa was largely healed (Figures 2E–2E′). Thus, DSS-induced injury causes early upregulation of *Ly6a* and the later emergence of a double-positive (*Ly6a+ Tnfr2+*) crypt population.

To assess whether *Tnfr2* positive cells can have a proliferative signature, we examined the correlation between *Tnfr2* and *Mki67* transcripts (Figures 2F–2J). In the affected region, at d 6, proliferation was reduced (Figure 2G), whereas at d 9–14, *Tnfr2* probes labeled the entire regenerative crypt structure, including the basal domain where *Mki67+* (proliferative) cells were primarily located (Figures 2H and 2I). In the unaffected area only *Mki67* expression was found (Figures 2F–2J). Thus, *Tnfr2* was expressed in cells of both proliferative and non-proliferative phenotype. We next examined whether differentiated cell fates could be found within the *Tnfr2+* cell population. Intriguingly, although *Muc2* (goblet-like) or *Reg4* (deep crypt secretory) staining was not found within *Tnfr2+* cells at d 9 (Figures 2M, 2P, 2S, and 2V; stars), by d 14, *Tnfr2+* cells were co-localized with *Muc2* and *Reg4* in regenerative crypt clusters (Figure 2N, 2P, 2T, and 2V; arrows). Hence, the late induction of *Tnfr2* transcript in regenerative crypts is correlated with the subsequent reduction of *Ly6a* expression and restoration of secretory cell differentiation in the injured epithelium.

### Colonic epithelial ablation of Tnfr2 disrupts tissue repair in acute injury

A simple model suggested by the temporal and spatial expression patterns of *Tnfr2* during wound repair is that TNFR2 functionally bridges the phenotypic change from relatively undifferentiated (regenerative) *Ly6a+* epithelium to differentiated homeostatic-like epithelium. To test the functional role of TNFR2 in colonic crypt regeneration in DSS-induced injury, we used mice with specific TNFR2 ablation in the intestinal/colonic epithelium. Mice harboring loxP-flanked *Tnfr2* alleles (Tnfr2^flox/flox^)^31^^,^^32^ were crossed with mice expressing Cre recombinase in the intestinal epithelium (*Vil1*-Cre).^33^ Analyses were performed within litters that contained both putatively “wild-type” mice (Tnfr2^flox/flox^) and mice with epithelial-specific deletions (*Vil1*-Cre; Tnfr2^flox/flox^). To confirm the loss of TNFR2 protein in colonic epithelium in *Vil1*-Cre; Tnfr2^flox/flox^ mice, we stained single-cell suspensions of digested colonic mucosa from *Vil1*-Cre; Tnfr2^flox/flox^ mice, whole-body *Tnfr2*^*−/−*^ mice, Tnfr2^flox/flox^, and WT mice, with anti-EpCAM and anti-TNFR2 antibodies. As shown in Figures S1A–S1A′, and consistent with targeted deletion, the levels of TNFR2 expression in EpCAM^+^ (epithelial) cells were reduced in *Vil1*-Cre; Tnfr2^flox/flox^ and *Tnfr2*^*−/−*^ mice compared to control genotypes. Similar results were obtained on RT-qPCR analysis.

**Figure 3:**
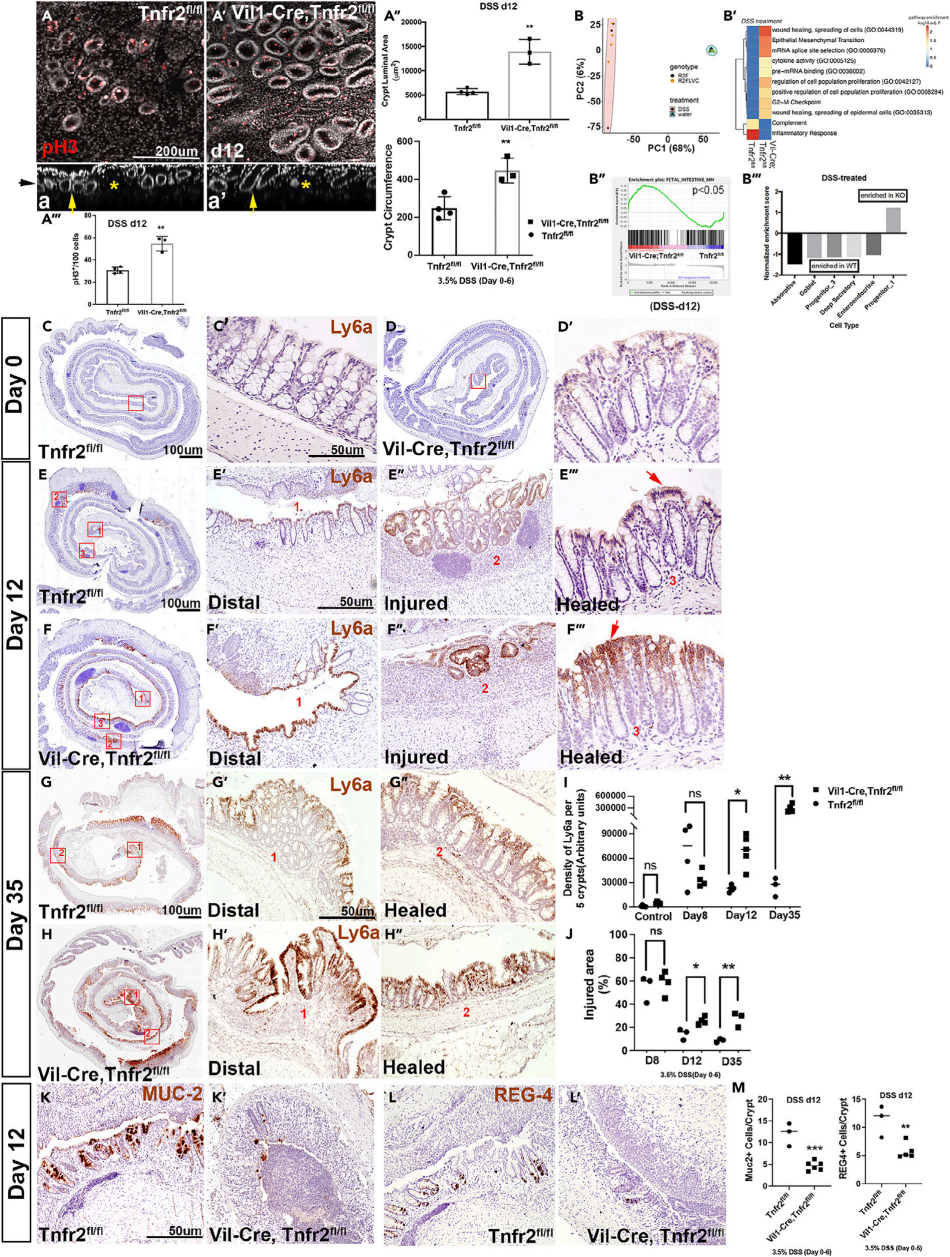
Colonic epithelial ablation of Tnfr2 disrupts tissue repair in acute injury (A) whole- mount images of distal colon at day 12 after DSS exposure was shown in *Vil1*-Cre; Tnfr2^flox/flox^ and Tnfr2^flox/flox^ mice (8weeks). TNFR2-deficient crypts labeled with methyl green had altered crypt morphology (A′) with enlarged lumen (A′-A″, um^2^; n = 3) and crypt circumference (A″, p = 0.008), and increased proliferation (A′-A‴; pH-H3, red; n = 4) compared to control (A-A‴; n = 4). (a-a’) Rotation of *en face* images in A and A′ showed enlarged lumen (arrow) and larger injured area (star) in *Vil1*-Cre; Tnfr2^flox/flox^ (a’) compared to Tnfr2^flox/flox^ (a). Black arrow shows the apical side in a. (∗∗p = 0.001; mean ± SD, unpaired t test). Scale bar: 200um. (B) Transcriptome sequencing was performed for sorted EpCAM^+^ (epithelial) cells from the injured regions of distal colon at day 12 after DSS exposure and day 0 (control) in *Vil1*-Cre; Tnfr2^flox/flox^ and Tnfr2^flox/flox^ mice. (B) PCA plot showed clusters of water-versus DSS-treated samples in both groups. (B′-B‴) Data analysis compared the differential activation of injury-induced pathways at day 12 between *Vil1*-Cre; Tnfr2^flox/flox^ and Tnfr2^flox/flox^ mice. It showed the enhancement of proliferation and regenerative signaling (B′), elevated fetal signaling (B″, p < 0.05), downregulation of differentiated cells, and enrichment of progenitor cells (B‴) in *Vil1*-Cre; Tnfr2^flox/flox^ compared to control. (C–F) *In situ* hybridization with *Ly6a* (brown) targeted probes showed elevated level of *Ly6a* in impaired (F′-F″) and healed (F‴; arrow) crypts of TNFR2-deficient epithelium at day 12 compared to Tnfr2^flox/flox^ (E-E‴, arrow) and control (day0, C–D). (C–F) The zoom-out views of Swiss roll colons were shown. Red squares with assigned numbers show the location of zoom-in images. Scale bars: 100 μm, 50 μm. (G and H) *Ly6a* transcript level remained high in distal colon and healed crypts at day 35 in TNFR2-ablated epithelium (H′-H”; brown) compared to Tnfr2^flox/flox^ (G′-G″). (G-H) The zoom-out views of Swiss roll colons were shown. Red squares with assigned numbers are showing the location of zoom-in images. Scale bars: 100um, 50um. (I) The density of *Ly6a* signal was significantly increased at day 12 (∗p < 0.02; n = 5) and day 35 (∗∗p = 0.001; n = 5) in *Vil1-Cre*; Tnfr2^flox/flox^ compared to Tnfr2^flox/flox^ mice. *Ly6a* density was not elevated at day 0 and 8 in TNFR2-ablated mice. (mean ± SD, unpaired t test). (J) Injured areas were significantly larger at day 12 (∗p < 0.02; n = 4) and day 35 (∗∗p = 0.001; n = 3), but not at day 8, in TNFR2-deficent epithelium compared to Tnfr2^flox/flox^ mice. (mean ± SD, unpaired t test). (K and L) Immunohistochemistry staining of MUC-2 and REG-4 proteins showed reduced number of goblet cells (K′, brown) and deep secretory cells (L′, brown) at day 12 in *Vil1*-Cre; Tnfr2^flox/flox^ mice compared to Tnfr2^flox/flox^ (K, L). (M) Statistical analysis showed the significant reduction of goblet cells (MUC-2) and deep secretory cells (REG-4) at day 12 in TNFR2-deficient epithelium. (∗∗p = 0.001, ∗∗∗p = 0.0005; mean ± SD, unpaired t test). Scale bar: 50 um.

Next, *Vil1*-Cre; Tnfr2^flox/flox^ and control Tnfr2^flox/flox^ mice were exposed to 3.5% DSS drinking water for 6 days, and colon tissues were collected from both groups on d 12 and d 35. Gross outcomes including body weight loss and hematopoietic (CD45^+^) cell infiltration, histologically scored immune cell infiltration, and neutrophil activation (MPO expression) were similar between epithelial TNFR2 knockouts and controls (Figures S1B–S1D). However, using a tissue clearing protocol^29^ to allow complete 3D rendering of colons labeled by the fluorescent methyl green dye,^34^ we found tissue morphological changes associated with epithelial loss of TNFR2. We found that intestinal epithelial-specific knockout animals exhibited enlarged crypt luminal area (3-fold; p = 0.004) and crypt circumference (2-fold; p = 0.008) and increased proliferation (phospho-histone H3 (pH-H3) a mitotic marker; p < 0.0001) (Figure 3A–3A‴; Figure S2). As complex morphogenesis occurs during injury and repair, in which crypts are eliminated (injury), and lumens of surviving crypts elongate and repetitively divide to form regenerated fields,^29^^,^^30^ we believe that these results are consistent with defective resolution of regenerative structural changes in the knockout.

To determine whether these morphological changes were linked to a regenerative transcriptional profile in TNFR2-deficient epithelium, we sorted EpCAM^+^ cells from *Vil1*-Cre; Tnfr2^flox/flox^ and Tnfr2^flox/flox^ DSS-treated and uninjured adult mice and performed bulk RNA-seq. The principal component plot (Figure 3B) demonstrated similar overall expression regardless of genotype in the uninjured (i.e., d 0) state. However, marked and variable changes in transcriptomes were found after treatment with DSS. For example, comparison of transcript expression profile between d 12 and d 0 (i.e., DSS-exposed vs. uninjured) in wild-type (Tnfr2^flox/flox^) mice revealed upregulation of pathways associated with metabolic regulation, ribosomal function, oxidative stress, TNF signaling, and focal adhesions at d 12 (Figure S3), consistent with DSS-induced inflammation and the wound healing response. Although the variability in samples after DSS treatment prevented assigning globally significant changes on the principal component plot; pathway enrichment analysis nonetheless suggested elevated regenerative (“fetal”) intestinal signaling, reduced inflammation, and increased proliferative signaling in *Vil1*-Cre; Tnfr2^flox/flox^ compared to Tnfr2^flox/flox^ colonic epithelium at d 12 (Figures 3B’–3B”). When compared against a reference atlas (Table S1) of single-cell transcriptomes of murine colonic epithelium,^35^ the knockout epithelium also showed reduced expression of markers of differentiated colonic epithelium and elevated expression of progenitor cell signaling (Figure 3B”’). Thus, the transcriptional data were consistent with increased regenerative signaling in TNFR2-knockout epithelium.

We next examined whether the loss of TNFR2 affected the spatial distribution and persistence of *Ly6a+* epithelial cells. Colon tissues of *Vil1*-Cre; Tnfr2^flox/flox^ and control Tnfr2^flox/flox^ mice were collected at d 0 (pre-injury), 8 (acute injury), 12 (early repair), and 35 (late repair). We analyzed the distribution of *Ly6a* transcript and quantified its expression using *in situ* hybridization. At d 0, we detected sparse labeling of epithelial cells, consistent with low-baseline levels of *Ly6a* expression (Figures 3C, 3D, and 3I). At d 8, its expression was induced in proximity to wounded regions similarly in both knockout and control genotypes and there were no significant changes in *Ly6a* density between groups (Figure 3I). However, at d 12, the labeling was markedly reduced in wild-type mice, whereas strong staining for Ly6a was still observed in the injured regions in knockout animals (Figures 3E, 3F, and 3I). At d 35, the overall colonic epithelial structure in wild-type animals had largely recovered albeit with slight residual expression of *Ly6a*^4^; however, in knockout animals, we continued to observe abnormal crypt structures that stained prominently with the *Ly6a* probe (Figures 3G, 3H, and 3I). Thus, the epithelial loss of TNFR2 is associated with the extended persistence of *Ly6a+* epithelial cells during the colonic injury-repair cycle.

We further analyzed whether loss of TNFR2 influenced other outcomes of epithelial status in DSS-injured colon. In “Swiss roll” histology of the colon, we quantified the fraction of the mucosa exhibiting crypt abnormalities or ulceration (Figure 3J). Wild type and TNFR2-deficient epithelium exhibited similar levels of injury at d 8, a time point consistent with maximal histological injury,^29^ but prior to the peak induction of TNFR2. However, at the later time points, the TNFR2-deficient epithelium remained proportionally more affected by injury. Moreover, the knockout epithelium had reduced the census of goblet and deep crypt secretory cells at d 12 (Figures 3K–3M). Together, these results suggest that loss of TNFR2 does not affect the initial magnitude of wounding or early repair but rather delays the later stages of wound healing and slows the return of secretory cell differentiation.

### TNFR2 knockout accelerates growth of colonoids

**Figure 4:**
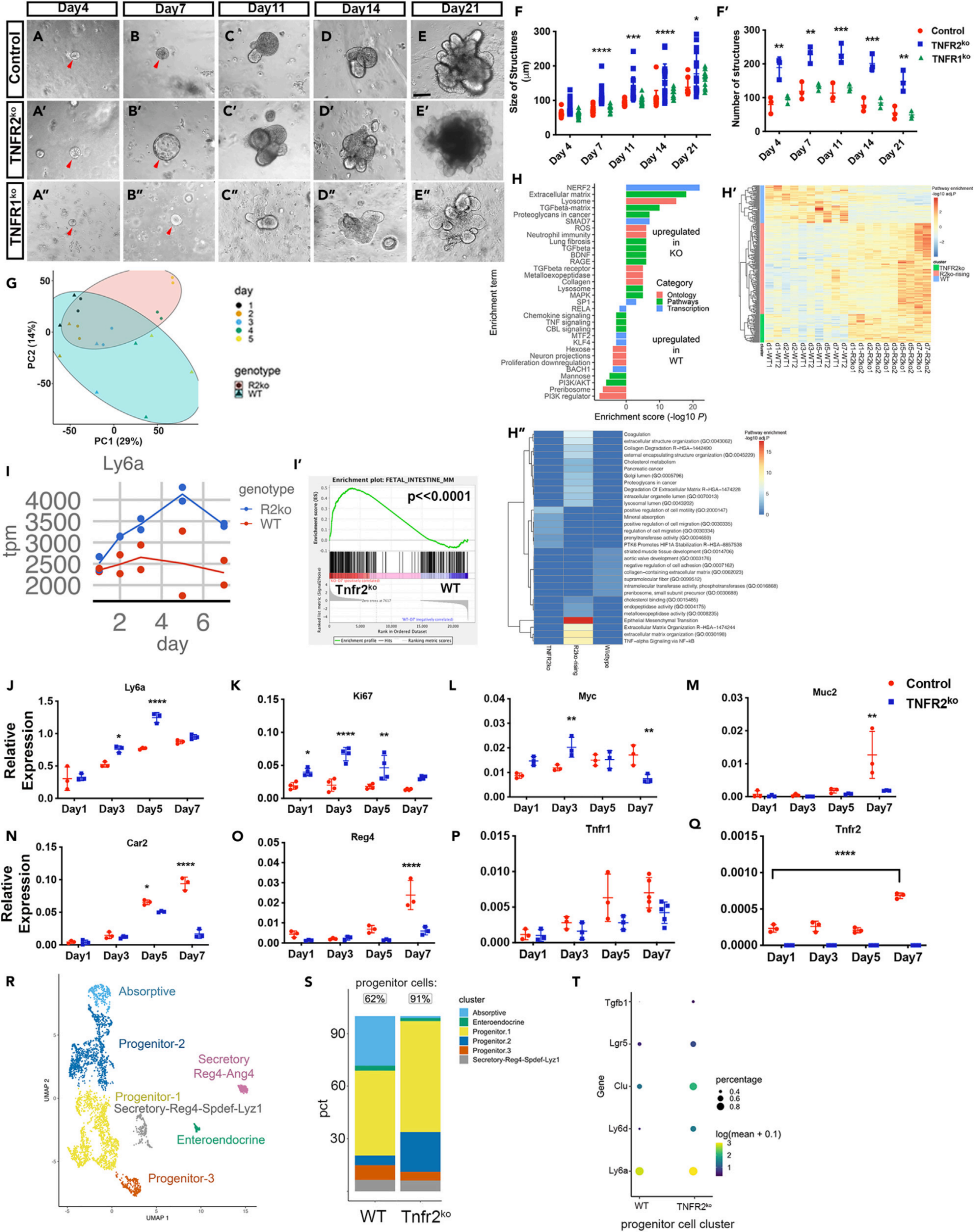
Transcriptomic characterization of TNFR2-deficient colonoids (A–E) The growth pattern of single-cell-derived colonoids is compared between whole-body *Tnfr2*^*−/−*^, whole-body *Tnf1*^*−/−*^, and wildtype (C57BL/6) mice (8weeks) after 4, 7, 11, 14, and 21 days *in vitro* (3 experimental repeats). *Tnfr2*^*ko*^-single cells were able to grow 1.5-fold faster than *Tnf1*^*−/−*^ and control colonoids over time. Scale bar: 100um. (F) The size of structures (μm) was significantly higher in *Tnfr2*^*−/−*^ colonoids compared to *Tnf1*^*−/−*^ and wildtype at day7 (∗∗∗∗p < 0.0001), day11(∗∗∗p = 0.0002), day14(∗∗∗∗p < 0.0001), and day21 (∗p = 0.02); and the number of structures (F′) were greater in TNFR2-deficient colonoids (day4–21) compared to *Tnfr1*^*−/−*^ and control. (∗∗p = 0.001, ∗∗∗p = 0.0005; mean ± SD, one-way ANOVA). (G–I) RNA-sequencing analysis of *Tnfr2*^*−/−*^ and wildtype colonoids were performed from day 1–7 post passage. (G) PCA plot showed cluster of WT vs. *Tnfr2*^*−/−*^ samples at different timepoints. (H) Changes in the activity of transcripts and pathways were analyzed in *Tnfr2*^*−/−*^ colonoids. (H′) Heatmap of aggregated gene expression was compared between WT, TNFR2^ko^-rising (genes that are elevated in the Tnfr2^ko^ specimens late in the time course) and TNFR2^ko^ (genes that are always higher in Tnfr2^ko^ specimens) clusters in WT and *Tnfr2*^*−/−*^ colonoids from day 1–7. (H″) The enrichment of pathways in WT, TNFR2^ko^-rising, and TNFR2^ko^ clusters was shown. (I) RNA-sequencing analysis of TNFR2-deficient colonoids showed the enrichment of fetal associated transcripts (I’; p≪0.0001) and upregulation of Ly6a at day 5–7. (J–Q) qPCR analysis of *Tnfr2*^*−/−*^ and wildtype colonoids showed the upregulation of *Ly6a* (J), *MKi-67* (K), *Myc* (L) and downregulation of *Muc2* (M), *Car2* (N), and *Reg4* (O) in *Tnfr2*^*−/−*^ colonoids. *Tnfr1* level remained unchanged (P) and *Tnfr2* was entirely absent (Q) in *Tnfr2*^*−/−*^ colonoids compared to control. (∗p < 0.02, ∗∗p = 0.001, ∗∗∗∗p < 0.0001; mean ± SD, unpaired t-test). (R–T) Single cell RNA-sequencing analysis of *Tnfr2*^*−/−*^ and wild-type colonoids were performed at 7 DPP. (R) Discrete clusters of progenitors (3 clusters), absorptive cells (1 cluster), and secretory cells (3 clusters) can be identified in the UMAP visualization. (S) The proportion of progenitor-like cells was increased to 91% of the recovered cells in *Tnfr2*^*−/−*^ compared to 62% in wild-type colonoids. (T) The elevation of *Ly6a*, *Ly6d*, *Tgfb1*, *Clu*, and *Lgr5* was shown in *Tnfr2*^*−/−*^ colonoids.

Although the loss of epithelial TNFR2 is correlated with the persistence of regenerative changes including *Ly6a* expression, epithelial hyperproliferation, and altered differentiation, it is possible that these effects are secondary to processes that govern the response to injury across multiple systems *in vivo*. To determine whether TNFR2 directly mediates changes in regenerative cells in a reductionist system, we tested whether TNFR2’s effects could be recapitulated in cultures containing colonic epithelial organoids (“colonoids”). We hypothesized that the growth dynamics of colonoid cultures in TNFR2’s absence might reflect continued regeneration and proliferation. We first established colonoid cultures from distal mouse colon and expanded them for >5 passages. We subsequently dissociated the cultures to single cells and compared the growth pattern of wild type, *Tnfr2*^*−/−*^, and *Tnfr1*^*−/−*^ derived colonoids, relying on the fact that only single cells with stem capability can re-form colonoids.^36^ We quantified the colonoid formation rate and the size and number of colonoids after 4, 7, 11, 14, and 21 days *in vitro*. We found that TNFR2-deficient colonoids establish more efficiently (2-fold; 3 experimental repeats) and grow 1.5-fold faster than their wild type or *Tnfr1*^*−/−*^ counterparts (Figures 4A–4E). The sizes of the overall structures (Figure 4F), as well as the number of buds (Figure 4F’), were higher for *Tnfr2*^*−/−*^ colonoids. This observation suggests that TNFR2 normally restricts epithelial proliferation and the organoid growth rate.

### Transcriptomic characterization of TNFR2-deficient colonoids

To determine the potential mechanism through which TNFR2 regulates epithelial dynamics in colonoids, we performed bulk RNA-seq analysis of WT and *Tnfr2*^*−/−*^ colonoid cultures at 1, 2, 3, 5, and 7 days post-passage (DPP). Unlike in Figures 4A–4F, in the following experiments, the organoids were not passaged to single-cell granularity. We first analyzed the dynamic changes in gene expression in WT organoids to obtain a normal sequence of organoid development. Previous single-cell data have shown that immediately after passage, small-intestinal organoids are in a *Yap1*-defined regenerative state for several days. Then, symmetry breaking and Paneth cell differentiation induce a cascade of budding and cellular differentiation.^37^ In the colonoid cultures here, gene set enrichment analysis (GSEA) showed enrichment of distinct pathways and cell phenotypes at early (DPP 1–3) versus late (DPP 5–7) time points after passage (Figure S4). In the early stage, the transcriptomes of colonoid cultures were dominated by proliferative signals. In contrast, in the late stage, the emergence of differentiation (e.g., glycosphingolipid biosynthesis, metabolism of carbohydrates) and increased interferon signaling were apparent. Thus, colonoid cultures exhibit dynamic regulation of overall transcriptional phenotype.

We next examined whether the pattern of gene expression changes was altered in *Tnfr2*^*−/−*^ colonoid cultures. We examined all genes that showed a significant genotype-associated difference and used hierarchical clustering to group them into modules. These modules circumscribed genes in *Tnfr2*^*−/−*^ cultures that were either: (1) persistently elevated, (2) persistently suppressed, or (3) specifically elevated in the late phase (Figure 4H’). In the late phase of growth (Figure 4G), *Tnfr2*^*−/−*^*-*associated pathways included upregulated TGFβ signaling, matrix metalloproteinases, and ECM components, consistent with matrix interactions that define early repair^4^ (Figures 4H–H”). In contrast, wild-type organoid cultures were relatively depleted of proliferation and enriched in chemokine and TNF-signaling pathways (Figure 4H). We noted that *Ly6a* increased in expression in *Tnfr2*^*−/−*^ colonoid cultures over time (Figure 4I). To determine whether the elevation of Ly6a was consistent with the regenerative (“fetal”) profile, we performed GSEA on the aggregated abundance values at 5–7 DPP and found significant enrichment of this profile in knockout colonoid cultures (p < 0.0001; Figure 4I’). Consistent with RNA-seq profiling, qPCR analysis of a validation cohort showed upregulation of regenerative and proliferative signaling (*Ly6a*, *Mki67*, and *Myc*), and downregulation of markers of differentiated cells including goblet cells (*Muc2*), differentiated colonocytes (*Car2*), and deep crypt secretory cells (*Reg4*) in TNFR2^−/−^ colonoid cultures compared to controls (Figures 4J–4O). The level of *Tnfr1* transcript increased over time but did not differ between genotypes (Figure 4P); thus, TNFR1 expression was not elevated to compensate for absence of TNFR2. TNFR2 was consistently expressed in wild-type organoid cultures and was entirely absent in knockout organoids (Figure 4Q).

To determine whether the bulk gene expression data correlated with changes in cell-type abundance, we performed single-cell RNA-seq analysis on WT and *Tnfr2*^*−/−*^ colonoid cultures at 7 DPP. We recovered 6,155 cells in the analysis, of which the majority (4,172 or 68%) were TNF-expressing *Krt8+* epithelial cells, and the remainder were colonic mucosal *Pdgfra+* fibroblasts (Figures S5A and S5B) expressing TGFβ. We identified 7 clusters of epithelial cells corresponding to progenitors (3 clusters), absorptive cells (1 cluster), and secretory cells (3 clusters) (Figure S5C). The proportion of progenitor-like cells was increased to ∼90% in the knockout condition, from ∼60% in the wild-type condition (Figures 4R–4T). Consistent with the bulk sequencing data, the expression of stem cell and regenerative genes including *Lgr5*, *Clu* (a “revival” stem cell marker,^38^ *Ly6a*, *Ly6d*, and *Tgfb1* were elevated in *Tnfr2*^*−/−*^ colonoids (Figure 4T). In summary, the impact of TNFR2 depletion in colonoid cultures recapitulates the persistence of *Ly6a* expression and reduced differentiation seen in a mouse colitis injury model. Although the presence of fibroblasts in the culture might partially contribute to mesenchymal-epithelial interactions underlying TNFR2’s effects, the combination of organoid studies and epithelial-targeted *Tnfr2* deletion *in vivo* nonetheless strongly suggests that TNFR2 acts directly on the epithelium after injury to reduce *Ly6a* expression, downregulate proliferation, and promote restoration of secretory cell differentiation, all hallmarks of colonic epithelial health and homeostasis.

## Discussion

Here, we show that the injury-associated induction of TNFR2 in colonic epithelium is critical for the downregulation of regeneration-associated transcriptional reprogramming and restoration of secretory cell differentiation in mice. In support of this model, we found that *Tnfr2* transcripts were elevated specifically in cells near the wound margin. In the DSS injury-repair model, which allows interrogation of different repair processes spread out over several days following acute colitis, peak *Tnfr2* expression occurred several days following initial induction of *Ly6a*, in overlapping crypt populations. Furthermore, loss of epithelial TNFR2 *in vivo* led to increased retention of *Ly6a* expression, persistent regenerative morphological changes, and delayed return of goblet and deep crypt secretory cell differentiation. These were likely direct effects, as they could be replicated *in vitro* in colonoids. Thus, a simple explanation for these findings is that the late upregulation of *Tnfr2* represents a mechanism that is utilized in *Ly6a+* cells to de-program them from the regenerative and hyperproliferative state.

The current model of intestinal epithelial repair posits that cells undergo reprogramming to a state resembling fetal epithelium. Activation of Yap/Taz may be critical for initiation of repair.^4^ In contrast, the signals that mediate the reversal of repair-associated reprogramming remain largely unknown, although retinoic acid signaling may represent one potential pathway.^37^ Here, we report that TNF signaling may represent another potential “reversal” pathway. Although the persistence of regenerative signaling in TNFR2-deleted mice might be predicted to induce faster wound healing, our results demonstrate the opposite, as epithelium-targeted knockout animals still exhibited ulcers late in the healing process. This would suggest that TNFR2’s functions are needed for proper healing of the mucosa or for the prevention of further injury and might be explained by TNFR2’s essential role in re-establishing the secretory cell population. Previous studies have shown that secretory cells are important for wound healing, as mucus serves as an important barrier against microbes, and goblet cells secrete pro-restitutive molecules such as trefoil factors.^39^^,^^40^^,^^41^^,^^42^ Our study thus identifies TNFR2 as an important regulator of wound repair.

Several upstream mechanisms may underpin the common function of *Tnfr2* in both the organoid and *in vivo* (DSS-treated mice) systems. Upregulation of the gene is observed in both contexts. In the *in vivo* mouse model, *Tnfr2* upregulation is correlated with time points of high-cytokine expression.^1^ Thus, cytokine elevations may be driving the observed effects. In fact, injury-associated reprogramming involves activation of interferon-responsive genes.^3^ However, similar transcriptional changes are seen in early organoid maturation and occur independently of interferon or immune-cell exposure.^37^ Thus, it may be that a broad set of pathways, perhaps associated with the release and detection of damage-associated molecular patterns during the breaking apart of large organoids, suffice as downstream triggers of reprogramming and *Tnfr2* upregulation, and can be activated by a variety of immune and non-immune injury stimuli.

TNFR2’s upregulation and functions can be viewed as part of an adaptive epithelial response to modulate cell dynamics, differentiation, and mucosal status at pivotal moments of cytokine/ligand elevation (inflammation), injury, and repair. This study reveals added functions for TNF beyond the mediation of inflammatory signaling and cell death. Although anti-TNF agents are commonly used to treat IBD,^10^^,^^11^ these agents exhibit a high-failure rate,^43^^,^^44^^,^^45^^,^^46^ which is often reflected by a lack of long-term mucosal healing.^47^ Genetic polymorphisms modulating expression of TNFR2 are also associated with response to anti-TNF in CD patients.^48^ These findings support the influence of TNFR2 in the therapeutic response to anti-TNF treatment. Future studies are needed to understand whether pharmacological TNF inhibition directly impairs wound resolution by suppressing epithelial TNFR2 signaling and conversely, whether stimulating TNFR2 could promote epithelial repair.

### Limitations of the study

TNFR2 did not exhibit significant effects on the metrics of inflammation used in this study. Dissecting the complex relationship between wound repair and inflammation is an important goal, but it may be more suitable to interrogate this in inflammation-driven models of colonic wounding such as the *Il10*^*−/−*^ model. In addition, deep analysis of mouse and human IBD datasets may help to define the range of cytokines that activate TNFR2’s expression and functions.

## STAR★Methods

### Key resources table

**Table:** 

REAGENT or RESOURCE	SOURCE	IDENTIFIER
**Antibodies**		

Rabbit anti REG4	Abcam	RRID: AB_ab255820
Rabbit anti MUC2	Santa Cruz Biotechnology	RRID: AB_sc-15334
Rabbit anti-pHH3 (Ser10)	Cell Signaling Technology	RRID: AB_9701S
Armenian Hamster anti-TNFR2(CD120b)	ThermoFisher Scientific	RRID: AB_MA5-32618
PerCP/Cy5.5 anti-CD326 (EpCAM)	Biolegend	RRID: AB_118219
FITC Rat anti-Mouse CD326(EpCAM)	Invitrogen	RRID: AB_11579180
Alexa Fluor 488 Rat anti-Mouse Ly-6A/E (Sca1)	Southern Biotechnology Associates	RRID: AB_175530
truStain fcX (Rat anti-CD16/32)	Biolegend	RRID: AB_101320
Streptavidin-PE	Biolegend	RRID: AB_405203
Alexa Fluor 555 Goat anti-Rabbit	ThermoFisher Scientific	RRID: AB_A27039
BV421 Donkey anti-Goat	JacksonImmuno Research	RRID: AB_705675147

**Chemicals, peptides, and recombinant proteins**		

Liberase TM	Sigma-Aldrich	5401119001
DNase I	Sigma-Aldrich	D5025
Y-27632	EMD Millipore	688000
CHIR99021	Stemgent	04-0004
IntestiCult	STEMCELL	06005
Matrigel	Corning	356237
DMEM/F12	STEMCELL	36254
Bovine Serum Albumin	Sigma-Aldrich	A9576
Dextran Sulfate Sodium (DSS)	MP Biomedicals	0216011090
3, 3 -diaminobenzidine (DAB)	Vector Labs	SK4105
Oxazolone	Sigma	862207

**Critical commercial assays**		

RNAscope.5 HD Reagent Kit Brown-Mm Kit	ACDBio	322371
RNAscope 2.5 HD Duplex Reagent Kit	ACDBio	322430

**Deposited data**		

Organoid bulk RNA-Seq	This paper	GSE157751 access token: mbelikcybfsnlwj
Organoid single-cell RNA-Seq	This paper	GSE230816 access token: yfilcswktnmtzad
Mouse colonic epithelium bulk RNA-Seq	This paper	GSE230329 Access token: yzwzgcggbnqdtmd

**Experimental models: Organisms/strains**		

C57BL/6	Jackson Labs	RRID:IMSR_JAX:000664
*Tnfr2*^*−/−*^ (B6.129S2-Tnfrsf1b^tm1Mwm^/J) #002620	Jackson Labs	RRID:IMSR_JAX:002620
*Tnfr1*^*−/−*^	Jackson Labs	RRID:IMSR_JAX:002818
*Il-10*^*−/−*^	Jackson Labs	RRID:IMSR_JAX:002251
*Vil1*-Cre (B6.Cg-Tg(*Vil1*-cre)1000Gum/J)	Jackson Labs	RRID:IMSR_JAX:21504
*Tnfr2*^*flox/flox*^	Institute Clinique de la Souris	RRID:EMMA ID: EM-05925

**Software and algorithms**		

FlowJo	FlowJo	10.4
ImageJ	NIH image	version 2.0.0-rc-67/1.52days
GraphPad Prism	GraphPad	7
Adobe Photoshop	Adobe	CC 2019
Kallisto/sleuth	Pachter Lab^49^	0.44.0
Monocle3	Cao et al.^50^	1.0.0

### Resource availability

#### Lead contact

Further information and requests for resources and reagents should be directed to and will be fulfilled by the Lead Contact, Brent Polk (dpolk@health.ucsd.edu).

#### Materials availability

This study did not generate new unique reagents.

### Experimental model and study participant details

#### Mice

All mice were housed in animal facility in accordance with regulations of the Institutional Animal Care and Use Committee (IACUC) (internal protocol#S21028) at University of California San Diego. Mice were co-housed for a week before setting the experiment and were euthanized with isoflurane prior to dissection. Strain and stock # of mice from Jackson Laboratory were as follows: C57BL/6 #000664, *Tnfr2*^*−/−*^ (B6.129S2-Tnfrsf1b^tm1Mwm^/J) #002620, *Il-10*^*−/−*^ #002251, *Tnfr1*^*−/−*^ #002818, Vil1-Cre (B6.Cg-Tg(Vil1-cre)1000Gum/J) #21504. *Tnfr2*^*flox/flox*^ (with a loxP-flanked TNFR2 exon 2; *Tnfrsf1b tm1c*) was originated in EMMA (EM#05925) and provided from ICS, Institut Clinique de la Souris, Illkirch/Strasbourg, France. Vil1-Cre; Tnfr2^flox/flox^ were generated by breeding of Vil1-Cre and *Tnfr2*^*flox/flox*^ mice at UCSD for >10 generations.

#### Dextran sulfate sodium (DSS) colitis injury model

Prior to the induction of colitis, mice (8 weeks; male and female) were trained to drink water only from the bottles for at least 3days. DSS colitis injury was induced in mice by administration of 3.5% w/v DSS (36–50 kDa M mass; MP Biomedicals) in distilled water for 6 days followed by regular drinking water. To validate the colonic injury, stool and body weight were measured regularly.

#### Oxazolone colitis

Oxazolone (Sigma #862207) was diluted in 50% ethanol to reach its final concentration of 6%. Mice (8 weeks; male and female) were anesthetized with isoflurane. 1mL-syringe mounted with a lubricated blunt cannula (ThermoFisher Scientific #202322) was used to instill 50 μl into the colon of C57BL/6 mice (8 weeks; male and female). Mice were inverted till end of recovery period.

### Method details

#### Histological processing

Euthanized mice were dissected, and the colon tissue was removed from the mice. The colon was opened longitudinally and washed with PBS to be clear from fecal contents. Flattered colon was fixed in 4% paraformaldehyde (PFA) overnight at 4°C. Paraffin embedding, OCT embedding, and histological staining such as hematoxylin and eosin (H&E) were performed according to standard protocols.

#### Immunohistochemistry

Immunohistochemistry was performed using standard protocols. Primary antibodies were incubated at 4°C overnight. Primary antibodies used were rabbit anti-pHH3 (Ser10) (Cell Signaling Technology #9701S, 1:500 dilution), rabbit anti-MUC2 (Santa Cruz Biotechnology #sc-15334, 1:500 dilution), rabbit anti-REG4 (abcam #ab255820, 1:500 dilution). Secondary antibodies (1:200 dilution) included a goat anti-rabbit Alexa Fluor 555 (ThermoFisher Scientific, #A27039).

#### Primary culture of colonic epithelial cells in matrigel

Cultures were established from the distal part of the colon from wildtype, Tnfr1^ko^, and Tnfr2^ko^ mice (8weeks; male and female). There were three experimental repeats, and for each experiment we used 3 mice per genotype. 2-mm^2^ tissue fragments were incubated with prewarmed digestion solution (0.2 Wunsch units/mL Liberase TM + 200 Kuntz units/mL DNase I in DMEM/F12 + 15 mM HEPES) for 30 min at 37°C with 180 rpm rotation. After vigorous pipetting and the passage of tissue through 100-μm-pore cell strainer, crypt units were washed with DMEM/F12 (STEMCELL, #36254) supplemented with 10% FBS, embedded in Matrigel (Corning, #356237) and cultured in IntestiCult organoid growth medium (STEMCELL, #06005). After 5 passages to dissociate organoids to single-cells, 40-μm-pore cell strainer was used. Isolated single-cells were washed with IntestiCult, imbedded in Matrigel (Corning) and cultured in IntestiCult medium supplemented with CHIR99021 (Stemgent #04-0004, 3 μM) and Y-27632 (EMD Millipore #688000, 10 μM, added for the first 2 days). Medium was changed every two days. The size and number of spheroids and budding structures were analyzed every day.

#### *In situ* hybridization

*In situ* hybridization was performed according to manufacturer’s instruction with the RNAscope.5 HD Reagent Kit Brown-Mm Kit (ACDBio #322371). The RNAscope Probe-Mm-Tnfrsf1b (#435941), Mm-Ly6a (#427571) from ACDBio was used. 10 min incubation with DAB (Vector Labs, #SK4105) was followed by counterstaining and mounting.

Duplex *in situ* hybridization was performed according to manufacturer’s instruction with the RNAscope 2.5 HD Duplex Reagent Kit (Chromogenic) (ACDBio #322430). The RNAscope Probe-Mm-Tnfrsf1b (#435941) with Mm-Ly6a-C2 (#427571-C2), Mm-mKi-67-C2 (#416771-C2), Mm-Muc-2-C2 (#315451-C2), or Mm-Reg4-C2(#409601-C2) from ACDBio was used.

#### RT-qPCR

Flow cytometric sorted cells were centrifuged at 1400 rpm. After removing the supernatant, the cells were suspended in 1mL Trizol overnight at −80°C. Using standard Trizol RNA extraction protocol, RNA was isolated from the sorted cells and precipitated with 800 ul Isopropyl alcohol. cDNA was prepared using the Verso cDNA Synthesis Kit from ThermoFisher Scientific (#AB1453B). The Quantitative PCR reaction was performed using the TaqMan Fast Advanced Master Mix (ThermoFisher Scientific #4444964) on a Bio-rad iQ5 thermocycler with 39-cycle repeats. Primer and probe oligonucleotides were obtained from IDT. Gusb and Hprt1 were used as reference genes (Wang et al., 2010). GUSB: (Mm.PT.39a.22214848), Hprt1: (Mm.PT.39a.22214828), Actb: (Mm.PT. 39a.22214843.g). Ly6a: (Mm.PT.58.49069476), Ki-67:(Mm.PT. 58.7871981), Myc: (Mm.PT.58.13590978), Muc-2: (Mm.PT. 58.29496069), Car-2: (Mm.PT.58.42151862), Reg-4: (Mm.PT.58.12037171), Tnfr1: (Mm.PT.58.13258743), Tnfr2: (Mm.PT.58.30148877).

qRT-PCR data were analyzed by using the comparative ΔΔCt method.^51^

#### Flow cytometry

8-week- old mice (male and female) were dissected and the colonic mucosal layer was detached from the muscle layer using fine forceps. In DSS colitis the injured colonic regions were identified under a stereomicroscope and separated from unaffected colon prior to downstream processing.

The 2-mm^2^ fragmented tissue was incubated with prewarmed digestion solution (0.2 Wunsch units/mL Liberase TM + 200 Kuntz units/mL DNase I in DMEM/F12 + 15 mM HEPES) for 30 min at 37°C with 180 rpm agitation. After vigorous pipetting and passage through 70-μm-pore cell strainer, tissue was washed with DMEM/F12 supplemented with 10% FBS, and then with HEPES-buffered saline^52^ supplemented with 0.5% BSA.

All subsequent washes and stainings were performed in HBS+0.5% BSA. The colonic cells were stained with 0.1 μM DAPI and blocked with a solution composed of 5% mouse/rat serum supplemented with mouse Fc block (rat anti-CD16/32 antibody, Biolegend “truStain fx” #101320) for 15 min at 4°C. Next, colonic cells were probed with primary antibodies (1:100 dilution) for 1 h at 4°C. After two washes cells were stained with a mixture of preconjugated (1:100 dilution) and secondary antibodies (1:200 dilution) for 30 min at 4°C.

PerCP/Cy5.5 anti-CD326 (EpCAM) (Biolegend #118219), Ly-6A/E (Sca1) rat anti-mouse Alexa Fluor 488 (Southern Biotechnology Associates, #175530), Armenian hamster anti-TNFR2(CD120b) (ThermoFisher Scientific #MA5-32618) primary antibodies, and donkey anti-goat Alexa Fluor 647 (Invitrogen #A21447), Streptavidin-PE (Biolegend #405203) secondary antibodies were used.

After washes, cells were analyzed on a BD LSR II. Ultracomp eBeads (ThermoFisher Scientific) with individual antibody were used as compensation controls. Flow cytometric data was analyzed using FlowJo. Gates were adjusted consistently between control and experiments through the software.

#### RNA sequencing

Total RNA from organoids and isolated murine colonic epithelium was submitted to SeqMatic (Fremont, CA) for stranded library preparation and sequencing on an Illumina NextSeq (high output run, 1 × 75). For organoid studies, two biological replicates were sequenced for each timepoint. Approximately 10 million reads were recovered per sample. Reads within FASTQ files were pseudoaligned and quantified using kallisto, and differentially expressed transcripts between wildtype and knockout conditions were identified using sleuth. Downstream analysis of data was performed with custom-written scripts in R.

Abundance files for input into Gene Set Enrichment Analysis were generated by summing and aggregate individual transcript-per-million (tpm) levels per transcript per gene. Each comparison of wildtype versus TNFR2-deficient samples was made over the Hallmark gene set and a lab-curated database of fetal and colonic cell-type specific genes. The q-value based on the normalized enrichment score obtained from GSEA was used to determine statistical significance of pathway enrichment. The gene members of the lab-curated database are shown in Table S1.^35^ Fetal genes are derived from the work of Mustata et al.^53^

Specific genes to each timepoint in organoid studies were identified by computing the timepoints of highest and second-highest expression. The log2-normalized expression ratio between these timepoints was calculated, and genes with a normalized ratio exceeding 1.0 were retained. The temporal expression pattern was then scaled and normalized in R, and hierarchical clustering applied with the cut-off chosen so that 2 clusters emerged (genes upregulated early and late). The results are shown in Table S2.

Genotype-associated genes in organoid studies were identified by selecting genes with a corrected p value <0.05 from the sleuth output wherein a linear model of expression was evaluated against the genotype factor. The temporal pattern of the selected genes was assigned among 3 clusters using hierarchical clustering. The pattern of the genes is shown in Table S3.

For single-cell RNA-Seq, single cell suspensions were obtained from colonic organoid cultures grown for 5 passages and isolated at 7 days after the last passage (7 DPP). Cells were lysed and barcoded with the 10X Genomics Chromium platform, pooled, and sequenced on the Illumina NovaSeq platform. UMI counts were obtained using cellranger. Downstream analyses were performed using monocle3. Cells with <100 UMI counts were filtered out. Abundance values per-cell were log-normalized by the total UMI count using the default methodology of monocle3. The top 100 principal components were used for dimensional reduction, and Leiden clustering at the default resolution was performed after uniform manifold approximation and projection (UMAP). Top markers of individual clusters were obtained using the top_markers() routine and was based on the calculation of Jensen-Shannon specificity scores, shown in Table S4. Mesenchymal cell clusters in the culture were identified by high expression of *Acta2* and were filtered out. Epithelial-mesenchymal doublets were identified by intermediate expression of epithelial (e.g., *Krt8*) and mesenchymal (e.g., *Acta2*) and were also filtered out. Epithelial cells were gated and subclustered to achieve the final cell-type assignments. Assignments were based on investigator-driven comparison with markers of known colonic epithelial cell-types.

#### Imaging

Image stacks were collected using a Zeiss LSM700 confocal inverted microscope with 5X, 10X or 20X lens with 1 Airy unit pinhole size. The air objectives were Plan-NeoFluar 10×/0.3 numeric aperture (NA), or Plan-Apochromat 20×/0.8 NA. Tiled images with 10% overlap were collected, and the matching of refractive index was performed with Zeiss software.

H&E images were acquired using a brightfield microscope.

### Quantification and statistical analysis

#### Statistical analysis

All statistical tests were calculated and graphed with GraphPad Prism 6. Significance was determined using mean ± SD, t-test or one-way ANOVA with Tukey’s post-hoc test. Quantifications were performed on images using built-in measurement functions in ImageJ.^54^

To analyze the density of a desired gene expression in Figures 1J and 2P and 2V, and 3I, we used the Integrated Density tool in ImageJ. For each histology image, the region of crypt was manually selected in the distal colon, and the Integrated Density tool calculated the sum of all stained pixels in a selected region. For each mouse, five contiguous crypts from a representative affected region were counted and the average of density value was presented.

To analyze the infiltration of immune cells at d 35 after exposure to 3.5% DSS in Figure S1C, H&E sections of both control and epithelial TNFR2 knockout groups were scored by Dr. Kay Washington based on Misra & parshad scoring system.^55^

To measure the length of injured area in DSS treated mice in Figure 3J, we used ImageJ. The length of affected regions was measured and normalized based on the whole length of each colon.

To analyze the size and number of structures in Figures 4A–4F′, we dissociated wildtype, *Tnfr2*^*−/−*^, and *Tnfr1*^*−/−*^ colonoids to single cells and seeded cells in 24-well plate for each genotype. From day 1–7, the number of survived single cells increased, cells grew and formed spheroids. After day 7, the number of structures did not change, spheroids grew and formed budding structures. After day 14, cells started to die, then the number of budding structures decreased. To measure the size of structures at different timepoint, we used ImageJ. The diameters of single cells, spheroids, and organoids were calculated and compared between experimental groups for each timepoint.

## Data Availability

• Sequencing data are deposited in the NCBI Gene Expression Omnibus (GEO) database and are publicly available as of the date of publication under the accession numbers GSE157751 (organoid bulk sequencing, reviewer access token: mbelikcybfsnlwj), GSE230329 (mouse colonic epithelium bulk sequencing, reviewer access token: yzwzgcggbnqdtmd), and GSE230816 (organoid single-cell sequencing, reviewer access token: yfilcswktnmtzad). These accession numbers are also listed in the key resources table. Microscopy data reported in this paper will be shared by the lead contact upon the request. • This paper does not report original code. • Any Additional information required to reanalyze the data reported in this paper is available from the lead contact upon the request.

## Supplemental information

Document S1. Figures S1–S5

Table S1. Reference gene lists used for gene set enrichment analysis of differential expression between TNFR2-deficient and wildtype cells, related to main Figure 3

Table S2. Differentially expressed genes and their pattern of upregulation during the maturation of colonic organoid cultures, related to main Figure 4

Table S3. Differentially expressed genes and their pattern of differential regulation between control and Tnfr2−/− colonic organoid cultures, related to main Figure 4

Table S4. Top markers of cell clusters obtained from single-cell analysis of colonoid cultures, related to main Figure 4
