# Supplementary material for: Colitis-induced upregulation of tumor necrosis factor receptor-2 (TNFR2) terminates epithelial regenerative signaling to restore homeostasis
Source: iScience. 2023 Sep 4;26(10):107829. doi: 10.1016/j.isci.2023.107829 (PMC10510063; doi:10.1016/j.isci.2023.107829)
Supplement: Document S1. Figures S1–S5 [file mmc1.pdf]

iScience, Volume *26*

Supplemental information

Colitis-induced upregulation of tumor necrosis factor receptor-2 (TNFR2) terminates epithelial regenerative signaling to restore homeostasis

Zohreh Sharifkhodaei, Cambrian Y. Liu, Nandini Girish, Ying Huang, Shivesh Punit, M. Kay Washington, and D. Brent Polk

Figure S1: Epithelial ablation of TNFR2 did not affect the severity of DSS-induced injury; Related to Figure 3

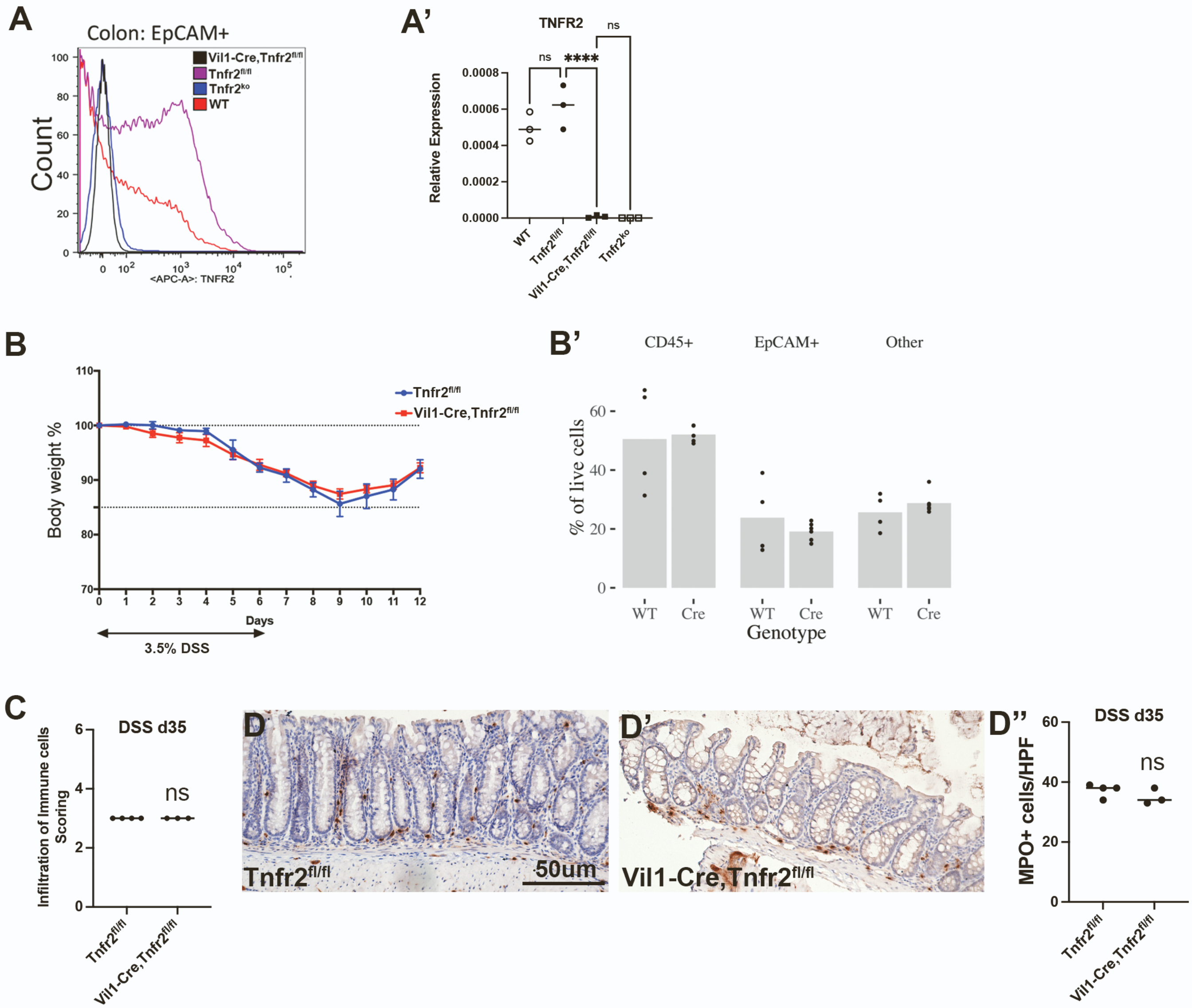

Figure S1: Epithelial ablation of TNFR2 did not affect the severity of DSS-induced injury
(A) Flow cytometric analysis showed a significant reduction of TNFR2 expression level in EpCAM+ (epithelial) cells in *Tnfr2*$^{-/-}$ and *Vil1-Cre*; Tnfr2$^{flox/flox}$ compared to Tnfr2$^{flox/flox}$ and Wildtype controls. (A') qPCR analysis of EpCAM+ sorted cells showed the loss of TNFR2 expression in *Vil1-Cre*; Tnfr2$^{flox/flox}$ compared to Tnfr2$^{flox/flox}$. **(B-D)** Data analysis showed no difference in the percentage of body weight loss (B), hematopoietic (CD45+) cell infiltration (B'), infiltration of immune cells (C; d35), and MPO expression (D-D"; d35) between *Vil1-Cre*; Tnfr2$^{flox/flox}$ and Tnfr2$^{flox/flox}$ (WT) after exposure to 3.5% DSS water. (****p=0.0001; mean±SD, unpaired t-test). Scale bar: 50um.

Figure S2: Epithelial-specific TNFR2^{ko} has a hyperproliferative signature; Related to Figure 3

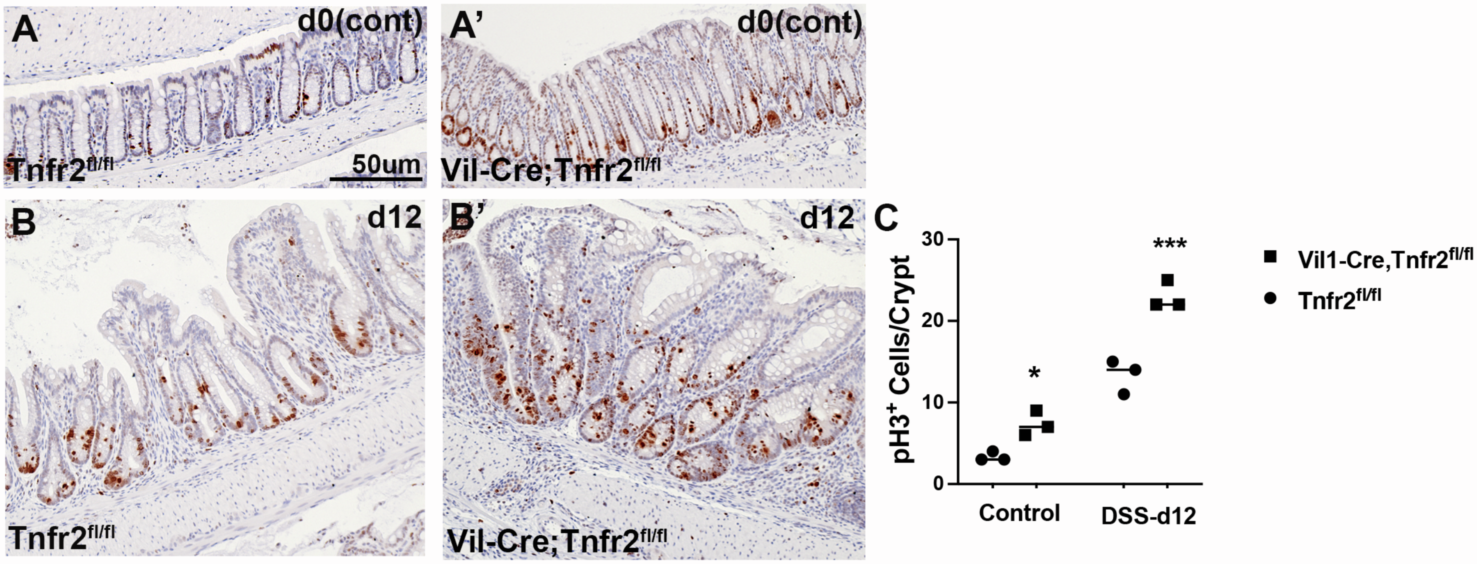

Figure S2: Epithelial-specific TNFR2ko has a hyperproliferative signature
(A-B) Epithelial ablation of TNFR2 showed increased proliferation (phospho-histone H3 (pH-H3); a mitotic marker) compared to TNFR2$^{fl/fl}$ (WT) at d0 (A, control) and d12 (B) after exposure to 3.5% DSS for 6 days. **(C)** Statistical analysis showed increased number of pH3+ cells per crypt in *Vil1-Cre*; Tnfr2$^{fl/fl}$ compared to Tnfr2$^{fl/fl}$ at d0 (control) and d12. (*p=0.01, ***p=0.0003; mean±SD, unpaired t-test). Scale bar: 50um.

Figure S3: Transcriptomic comparison of pathway enrichment between exp d12 and day 0 in control mice; Related to Figure 3

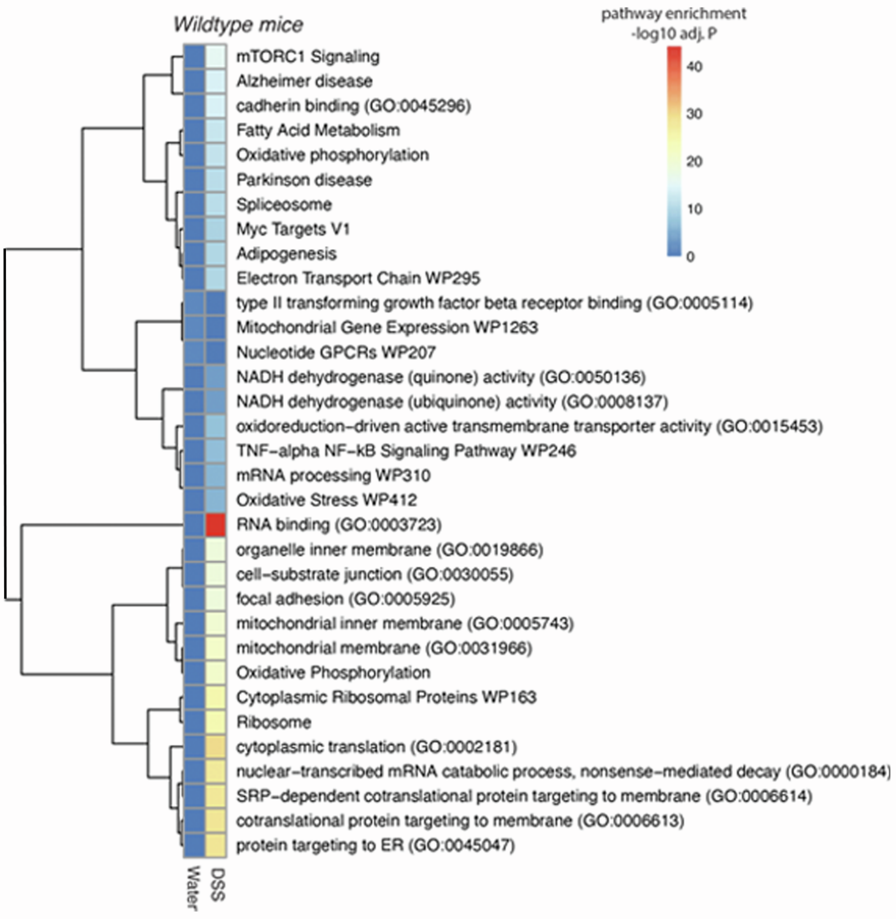

Figure S3: Transcriptomic comparison of pathway enrichment between exp d12 and day 0 in control mice

Transcriptome sequencing was performed for sorted EpCAM+ (epithelial) cells from the injured regions of distal colon at day 12 after DSS exposure and day 0 (control) in Tnfr2$^{flox/flox}$ mice. Comparison of transcript expression between day 12 and day 0 revealed upregulation of pathways associated with metabolic regulation, ribosomal function, oxidative stress, TNF signaling, and focal adhesions at day 12 in control mice.

Figure S4: Transcriptomic analysis of wildtype colonoids; Related to Figure 4

A

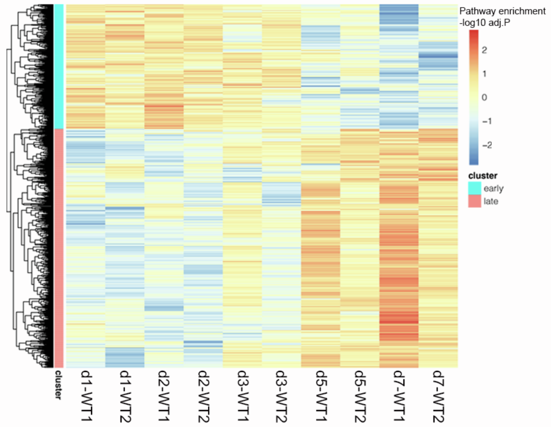

A'

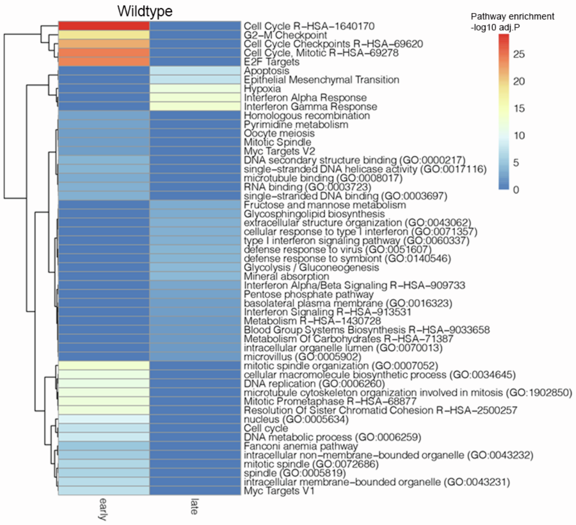

Figure S4: Transcriptomic analysis of wildtype colonoids
(A) Heatmap plot of individual gene progression was compared in early and late clusters in wildtype colonoids from day 1 to 7. (A') The enrichment of pathways in early and late clusters was shown in wildtype colonoids.

Figure S5: Single-cell RNA-seq analysis on WT and Tnfr2$^{-/-}$ colonoid cultures; Related to Figure 4

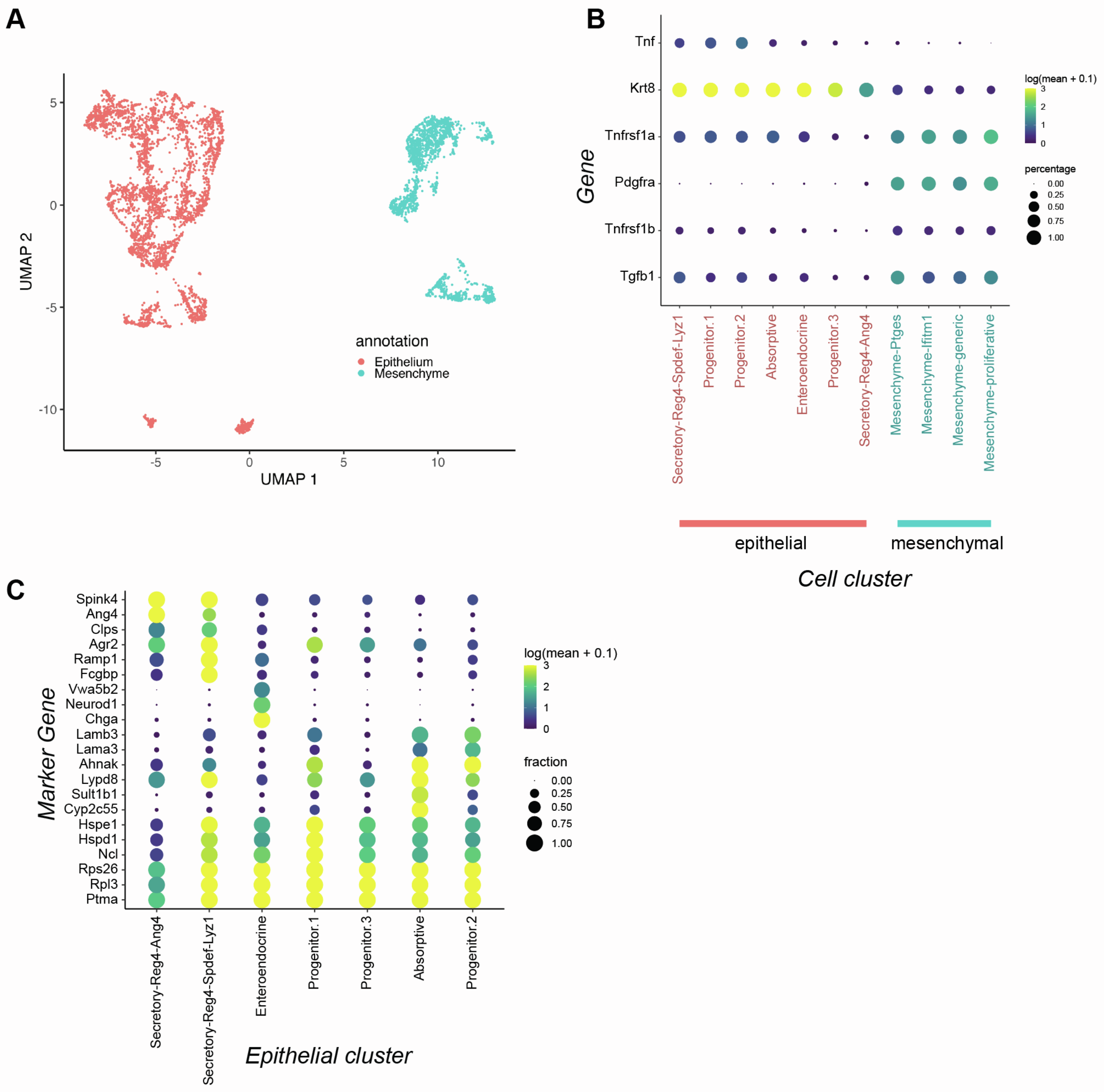

Figure S5: Single-cell RNA-seq analysis on WT and Tnfr2-/- colonoids
Sc RNA-seq analysis was performed on WT and Tnfr2-/- colonoids at 7 DPP. **(A-B)** 6,155 cells were recovered in the analysis, of which the majority (4,172 or 68%) were TNF-expressing Krt8+ epithelial cells, and the remainder were colonic mucosal Pdgfra+ fibroblasts expressing TGFb. **(C)** 7 clusters of epithelial cells corresponding to progenitors (3 clusters), absorptive cells (1 cluster), and secretory cells (3 clusters) were identified.
